# Supplementary material for: Hypergammaglobulinemia in treated and untreated people with HIV
Source: PLoS One. 2026 May 21;21(5):e0349405. doi: 10.1371/journal.pone.0349405 (PMC13193345; doi:10.1371/journal.pone.0349405)
Supplement: S1 Table — Ongoing infection or malignancy was defined as the presence of an opportunistic or non-opportunistic bacterial, viral or parasitic infection or active cancer in association with or within two weeks of blood sampling. n, number of diagnoses; CMV, cytomegalovirus; PML, progressive multifocal leukoencephalopathy; VZV, varicella- zoster virus. Nine out of 129 participants were diagnosed with more than one ongoing infection. (DOCX) [file pone.0349405.s001.docx]

|  | | *n* |
| --- | --- | --- |
| Pneumocystis jirovecii pneumonia | 50 | |
| Tuberculosis | 16 | |
| Syphilis | 11 | |
| Candida esophagitis | 7 | |
| Herpes zoster | 7 | |
| Bacterial pneumonia | 6 | |
| Toxoplasmosis | 5 | |
| Mycobacterium avium complex infection | 4 | |
| CMV infection | 3 | |
| Cryptococcal meningitis | 2 | |
| Cryptosporidiosis | 2 | |
| Chlamydia | 2 | |
| PML | 2 | |
| VZV meningoencephalitis | 2 | |
| Gastroenteritis | 1 | |
| Shigella gastroenteritis | 1 | |
| Campylobacter gastroenteritis | 1 | |
| Endocarditis | 1 | |
| Cerebral histoplasmosis | 1 | |
| Gonorrhea | 1 | |
| Cutaneus leishmaniasis | 1 | |
| Empyema | 1 | |
| Sepsis caused by Salmonella spp | 1 | |
| Sepsis caused by Streptococcus pneumonia | 1 | |
| Sepsis caused by Group B streptococcus | 1 | |
| Cystoisosporiasis | 1 | |
| Parotitis | 1 | |
| Upper respiratory infection | 1 | |
| Neuroborreliosis | 1 | |
| Anal abscess | 1 | |
| Non-purulent coxarthritis | 1 | |
| Hematological malignancy | 3 | |
| Total | 139 | |
