# Supplementary figures and images for: Hypergammaglobulinemia in treated and untreated people with HIV
Source: PLoS One. 2026 May 21;21(5):e0349405. doi: 10.1371/journal.pone.0349405 (PMC13193345; doi:10.1371/journal.pone.0349405)

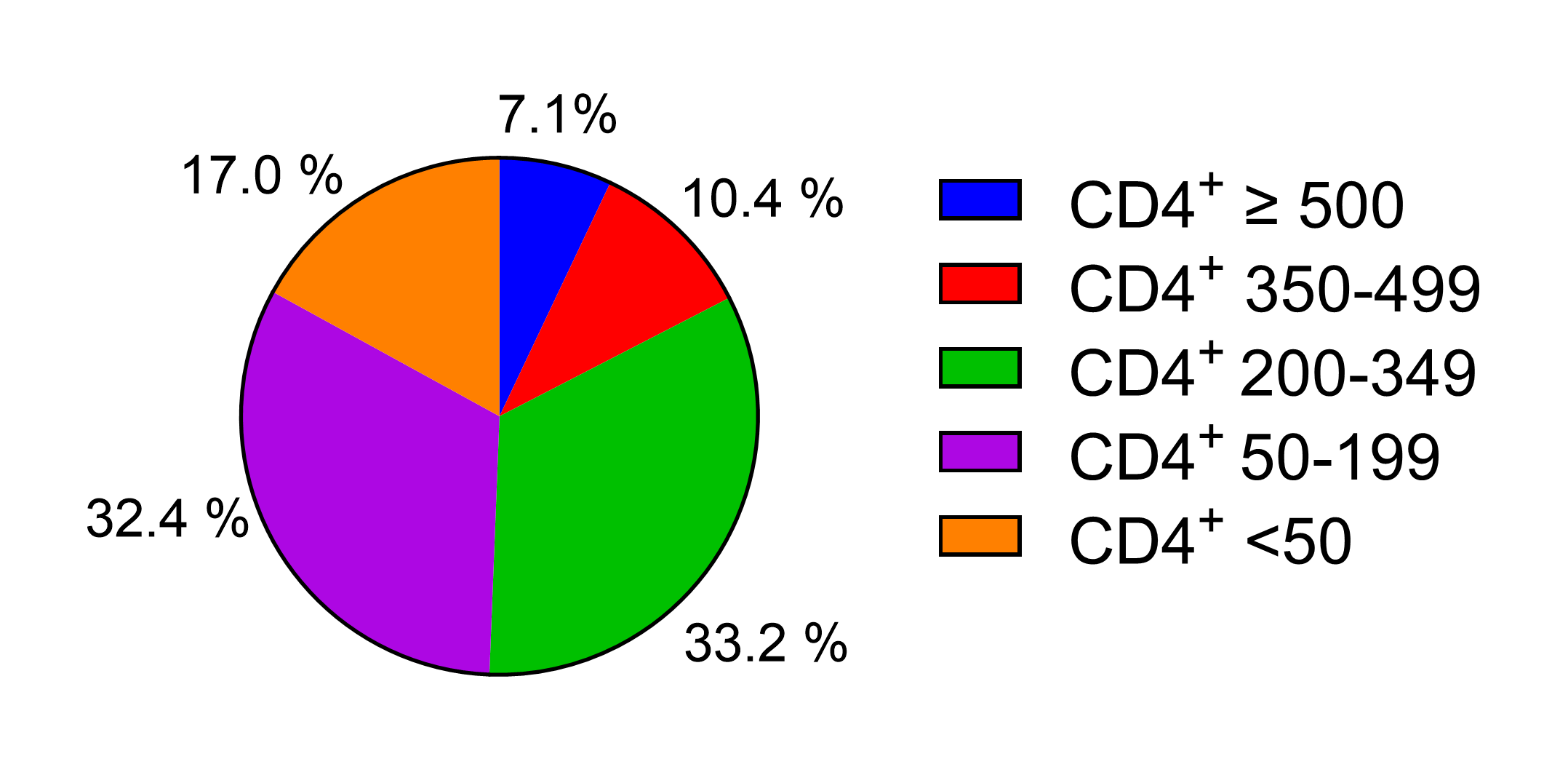

Supplement: S1 Fig — ART; antiretroviral therapy with plasma HIV-RNA < 50 copies/mL for ≥ 6 months. Data on CD4+ T cell nadir was missing for 26 out of 267 participants (9.7%). (TIF) [file pone.0349405.s002.tif]

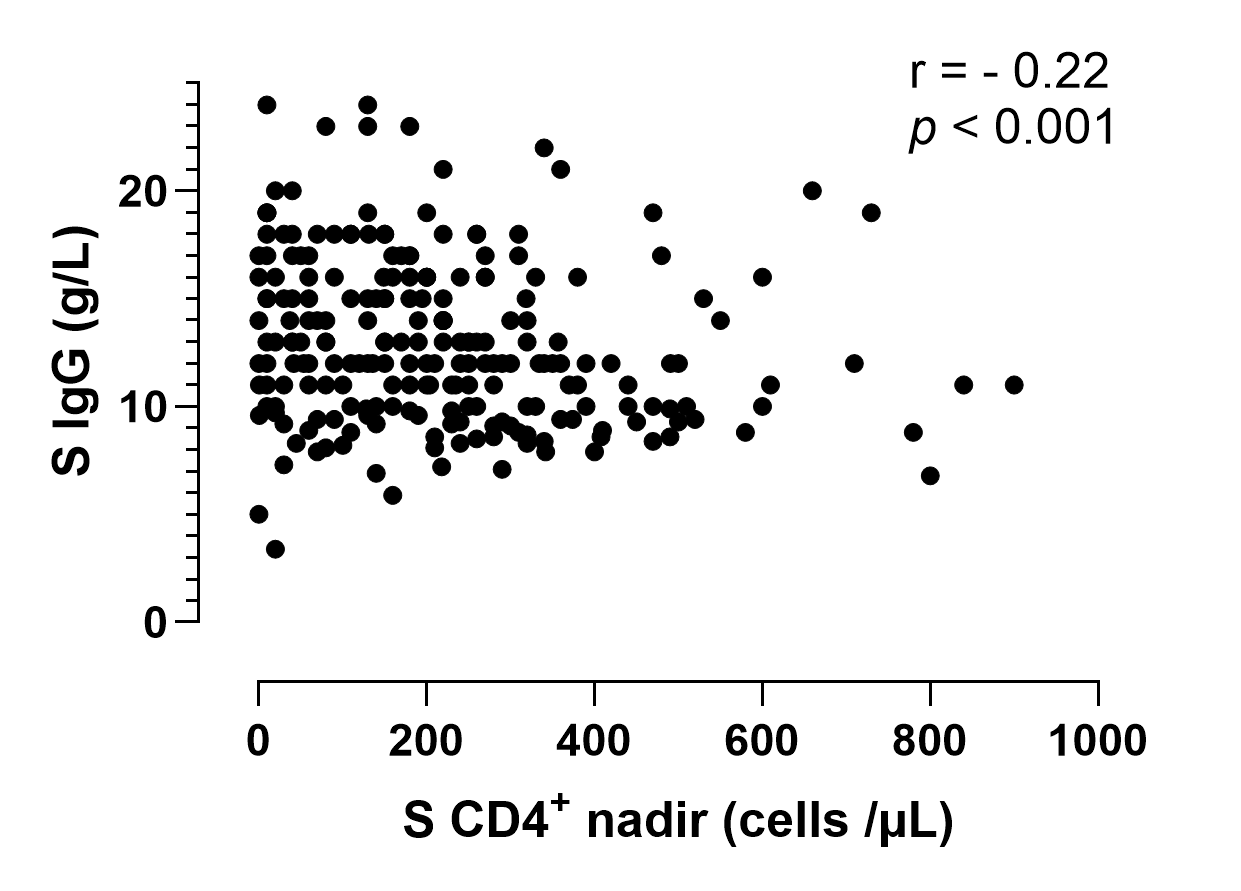

Supplement: S2 Fig — IgG, immunoglobulin G; ART; antiretroviral therapy with plasma HIV-RNA < 50 copies/mL for ≥ 6 months. Data on CD4+ T cell nadir was missing for 26 out of 267 participants (9.7%). IgG reference interval: 6.8 − 15.0 g/L. (TIF) [file pone.0349405.s003.tif]
