# Supplementary material for: Hypergammaglobulinemia in treated and untreated people with HIV
Source: PLoS One. 2026 May 21;21(5):e0349405. doi: 10.1371/journal.pone.0349405 (PMC13193345; doi:10.1371/journal.pone.0349405)
Supplement: Attachment — Submitted filename: Respons to Reviewers.docx [file pone.0349405.s004.docx]

**Response to reviewers**

“Hypergammaglobulinemia in treated and untreated people with HIV”

First, we wish to thank the two reviewers for their interest in our work and their constructive comments on the paper. We have revised the manuscript accordingly, and we address each comment below.

**Reviewer 1**

*Major comments*

*Q1: The manuscript alternates between describing the study as retrospective and prospective. Please clarify the study's exact design and use consistent terminology in the Abstract, Methods, and Results sections. If this is a retrospective analysis of prospectively collected cohort data, state this clearly. In lines 97–98, clarify whether some participants contributed multiple samples and how this was addressed in the analysis. If repeated measures were used, consider mixed effects or Generalized Estimating Equations (GEE) models, or select one sample per participant with sensitivity analyses. Additionally, in line 99, replace* *“One hundred sixty-six” with numerals.*

A1: Thank You for this valuable comment. We have clarified that the study is a cross-sectional analysis of prospectively collected data, and the term “retrospective” has been removed throughout the manuscript.

For the group comparisons in Table 2, no participant contributed more than one sample; each sample represents a unique subject, either in the ART group or one of the untreated groups, thus all samples are independent. This has been clarified in the Definitions section (lines 121-123) and in the captions of Table 1 and 2 (lines 189-192 and 219-222). Prompted by this remark, we also revised Figure 2, dividing the HIV-positive samples into ART-treated and untreated groups. Finally, “one hundred sixty-six” has been replaced with numerals.

*Q2: The definition and ascertainment of primary HIV infection (PHI) require more detail. While PHI is defined as within 12 months of infection by the authors, the criteria used to determine this are not described. Specify whether the diagnosis was based on documented seroconversion, RNA+/Ab status, p24 antigen detection, or patient history, and* *address the possibility of late presenters being misclassified as PHI. Additionally, if authors could consider adding all category definitions in the article to a dedicated “Definitions” subsection.*

A2: The diagnosis of primary HIV infection (PHI) was based on a combination of clinical symptoms and laboratory confirmation through seroconversion, nucleic acid testing or ELISA results. This information has been added to the Definitions section (lines 110-112). The combined use of clinical and laboratory data minimizes the risk of misclassification of infection stage. Further, definitions for all study categories have now been consolidated under a dedicated “Definitions” subsection, as suggested.

*Q3: Given that the data span from 1985 to 2023 and measurements were performed on different assay platforms, describe any calibration or harmonization procedures and assess whether platform or sampling era confounded the results. Sensitivity analyses restricted to uniform assay periods would strengthen the findings.*

A3: Thank You for raising this important point regarding the change in analytical methods for IgG measurement during the study period. When the analytical instrument was replaced, a comprehensive validation was performed, including verification of the reference interval. The laboratory has also participated continuously in two external quality assurance programs (Equalis and INSTAND). According to available validation and external quality control data, the transition from nephelometry to immunoturbidimetry did not result in any clinically relevant differences in IgG concentrations. This information has been added to the Laboratory methods section (lines 143-145).

*Q4: The authors state that the control group includes both PrEP and non‑PrEP individuals. It would be great if authors consider presenting subgroup analyses or adjusting for PrEP status in all control comparisons to avoid bias in defining what we may call “normal” reference ranges.*

A4: While HIV-negative controls using PrEP showed higher concentrations of β2-microglobulin and neopterin when compared to HIV-negative controls not using PrEP, there was no significant difference in IgG levels between these groups. This has now been stated in the Results section (lines 204-206). The observed biomarker differences between PrEP and non-PrEP controls have been described previously (Robertson et al, references 28 and 30) and this has now been addressed in the Results (lines 262-265) as well as in the Discussion (lines 351-355. We have also performed a subgroup analysis comparing HIV-negative controls with and without PrEP in all control comparisons and the overall results remained unchanged (lines 206-207).

*Q5: The statistical analysis involves numerous pairwise tests without adjustment for multiple comparisons. Apply false discovery rate or family‑wise error control and report effect sizes (e.g., median differences with 95% CIs) alongside p‑values. Clarify the handling of non‑normal data and log‑transformed variables.*

A5: In table 2, 32 pairwise tests were performed. Using Holm-Bonferroni, which is still conservative due to correlated tests, to correct for multiple inference does not change the status of any of the nominally significant tests. We agree that effect sizes are generally important, however, in this context we believe including them would make the table less readable. For this reason, we decided not to include these data. Regarding data distribution, non-normally distributed variables were log-transformed prior to analysis, as stated in the Statistical methods section (lines 165-175).

*Q6: Missing data for key biomarkers are noted in Table 1 and 2 captions. Authors could take time to state explicitly how missing values were handled, for instance, excluded, imputed, and assess whether the missingness was random.*

A6: Thank You for this comment. Regarding the missing data for inflammatory biomarkers (neopterin, β2-microglobulin, albumin), these were missing by random, e.g., when the remaining blood volumes were insufficient for the required assays. Hence, no systematic error was identified. Missing data were excluded pairwise in the statistical analyses. This information has been added to the captions of Table 1 and Table 2 (lines 193-194 and 224-225, respectively).

*Q7: In Table 2, summing the figures in column (n) gives a total of 892, which exceeds the stated sample size. Please clarify the actual study sample size and explain any discrepancies.*

A7: Thank You for noting this. The HIV-negative control group (*n* = 71) was subdivided into participants using PrEP (*n* = 45) and not using PrEP (*n* = 26). Thus, the total number of samples (*n* = 821) is correct. To clarify this, we have right-aligned the participant number for the HIV-negative subgroups (with and without PrEP) in Table 1 and Table 2. In addition, information on the total sample size has been added to the table legends (lines 189-192 and 219-222).

*Q8: Figure 3 is mentioned in line 244 but is not included in the manuscript. Ensure all referenced figures are present, correctly numbered, and match their in‑text citations, and also the numbering must be sequentially ordered for both Tables and Figures in the article.*

A8: Figure 3 was submitted along with the manuscript, but seems to be missing in the file presented to Reviewer 1.

*Q9: Some discussion statements, particularly those around lines 341-353, are not fully supported by the presented data. Revise to ensure interpretations are directly linked to results and acknowledge limitations in small subgroups.*
A9: Thank You for this comment. It appears there may have been a line displacement, as lines 341-353 contained the supporting figure captions rather than discussion text. We would appreciate clarification regarding which specific part(s) of the discussion Reviewer 1 is referring to, so that we can address the concern appropriately.

*Q10: Lines 41-43: The interpretation of inflammatory biomarker data could be expanded further. The statement “About one‑third of ART‑suppressed participants have elevated neopterin and β2‑microglobulin.” If authors could quantify the overlap with elevated IgG and explore predictors of persistent hypergammaglobulinemia using adjusted models, this could help with clarity on the data presentation in the article.*

A10: We assume that the Reviewer is referring to lines 228-230 in the previous manuscript version. Instead of quantifying the overlap, we would like to refer to Figure 3, which appears to be missing from the manuscript provided to Reviewer 1. Here, correlation coefficients were explored for IgG regarding HIV-RNA, CD4^+^, neopterin, beta-2-microglobulin, albumin, and age in untreated and ART-suppressed HIV-positive participants.

*Q11: The Methods section and data analysis descriptions are not sufficiently detailed. Please expand these to clearly explain the analytical approach, including any statistical tests, adjustments, and handling of repeated measures.*

A11: Thank You for this valuable comment. The statistical section has been rewritten.

*Q12: The Swedish epidemic cohort reflects a male predominance, and the authors appropriately noted this. However, the finding that women had significantly higher IgG levels is interesting. Could this be explored further? Was this difference consistent across all CD4 strata and treatment groups? A brief comment on this would add depth.*

A12: We agree with the Reviewer that the gender differences in IgG levels warrant further discussion. We have therefore expanded the analysis of IgG levels in men and women within the untreated groups, and this is commented on in the Results section (lines 241-246). Gender differences in IgG levels in the general population as well as among HIV-negative controls have been addressed in the Discussion section (lines 329-336). Additionally, Figure 2 has been modified to present HIV-positive samples divided into ART-treated and untreated groups.

*Q13: Given the numerous factors influencing IgG levels (sex, treatment status, CD4 count, and inflammatory markers), a multivariate regression analysis would be highly valuable. This would help determine the independent predictors of IgG levels (and persistent hypergammaglobulinemia on ART) while controlling for other variables. The current univariate analyses are informative but could be significantly strengthened by a multivariate model.*

A13: We have performed a multivariable analysis, but did not include neopterin and β2-microglobulin, since we do not consider them as causal determinants to IgG, but rather correlated parallel outcomes to IgG that misleadingly attenuate the impact of the other predictors when included. The results from the multivariable analyses are reported in the Results section (lines 207-211).

*Q14: Lines 314-322: The discussion on clinical implications is somewhat speculative. The suggestion that IgG could be a surrogate marker for B-cell dysfunction is interesting. Could the authors be more specific? For instance, in which clinical scenarios might measuring IgG be most useful (monitoring patients with incomplete immune reconstitution despite ART)? Also, please clarify the statement on lines 301-302; low albumin was only found in the OIM group, so it cannot be a general marker of HIV-related inflammation but rather of acute concomitant illness.*

A14: We have revised the final sentence to make the interpretation of persistent hypergammaglobulinemia of ART-suppressed participants less speculative (lines 385-387).

We also agree that the observed hypoalbuminemia in the OIM study group is likely related to the concomitant infection or malignancy, rather than to HIV-infection itself. This has been clarified in the Discussion section (lines 355-357).

*Minor comments*

*Q1: Under the Abstract section, define all abbreviations at first use, for instance “human immunodeficiency virus (HIV)” and “immunoglobulin G (IgG)”. Apply this consistently for all abbreviations throughout the manuscript, including in tables and figures*

A1: All abbreviations have now been defined in the manuscripts as well as in the figure and table legends.

*Q2: Line 133: "guidelines existing at the time of inclusion". Please specify the core principle, for instance, if authors considered a combination of at least two drugs from different classes. Additionally, what would be of value if guidelines could be cited.*

A2: We have added citations for past and present Swedish treatment guidelines (lines 114-118).

*Q3: Figure 2: The figure is helpful. Consider adding the p-values for the significant comparisons (HIV+ untreated and ART) directly to the figure or its legend for clarity.*

A3*: P* values have been added. Further, we have chosen to divide the HIV-positive group into treated and untreated participants, since 166 participants provided two samples and hence were included in both groups.

*Q4: Figure 3: The axis labels for Figure 3D should be "S-β2-microglobulin (mg/L)".*

A4: Hyphens have been added consistently in tables and figures.

*Q5: Line 275: "in 45 % and 37 %". This is fine, but for consistency, you could use the "%" symbol throughout (e.g., 45%).*

A5: The gaps between numbers and percentage signs have been removed.

*Q6: Line 311: "mean age of 42 years". Could this be a typo in the results (Table 1) report? Please, authors, report the "median" instead.*

A6: “Mean” has been changed to “median”. Moreover, we have reported the median age for untreated and treated participants, respectively.

*Q7: Correct unit errors, for instance, (“ml/L” to “mg/L) for β2‑microglobulin, unless perhaps there were some conversions applied and typographical inconsistencies, for instance, “IgG -levels” to “IgG levels” (line 288) and standardize HIV‑1 vs HIV usage.*

A7: These inconsistencies have been corrected.

Q8: *Ensure all tables and figures have self‑contained legends that define abbreviations, state reference limits, and specify comparison frameworks. Add 95% CIs to medians in tables where possible.*

A8: The legends for all tables and figures have been reviewed to ensure they are self-contained, with definitions of abbreviations and clear descriptions of comparisons frameworks. Reference intervals have been added to the legends of Table 2, Fig 2, Fig 3 and S2 Fig. We chose not to add 95 % CI, as we believe presenting median (IQR) values provide a clearer and more accessible summary of the data.

*Q9: Where qualitative terms like “weak correlation” are used, include numeric rho and p‑values in text or figure captions.*

A9: Numeric rho and *P* values are shown in Figure 3, which unfortunately seems to be missing for Reviewer 1.

*Q10: When comparing results to other countries, provide possible explanations for differences (e.g., ART coverage, prophylaxis use, cohort characteristics) rather than stating comparability without context.*

A10: No comparisons between countries have been made in the manuscript.

*Q11: Check that all data in the text match the values in tables and figures and review the manuscript for typographical and formatting errors.*

A11: The manuscript has been reviewed for typographical and formatting errors.

*Q12: If applicable, include an accession or URL for the Swedish National Data Service in the Data Availability statement.*

A12: The anonymized dataset for the present study has been uploaded to the Swedish National Data Service with restricted access due to participant confidentiality. The DOI is stated in the Data availability statement, line 391.

**Reviewer 2**

*Major comments*

*Q1: In lines 99-101, the authors mention that 166 participants provided samples twice and were thus included in both treated and untreated groups. This creates a significant issue of non-independence between the ART and the various untreated CD4 strata groups. The statistical comparisons between these groups are potentially confounded by this repeated sampling. The authors must clearly address this as a key limitation in the discussion. The statistical analysis should be clarified; were comparisons between groups treated as independent? If so, this is a methodological weakness. Consider using statistical methods that account for paired/repeated measures for these specific comparisons or focusing more on cross-group trends rather than direct pairwise tests between these non-independent groups.*

A1: Thank You for this valuable comment. All samples were analyzed independently, and no participant contributed more than one sample to any of the comparisons. No other HIV-positive participants than those in the ART-group received antiretroviral therapy. In the group comparisons in Table 2, each sample represents a unique subject, either from the ART group or from one of the untreated groups. Importantly, no direct comparisons were made between the different HIV-positive study groups, but only between each HIV-positive study group and the HIV-negative control group*.* This has now been clarified in the Methods section (lines 121-123) and in the legend of Table 2 (lines 222-223).

*Q2: In the methods section, Lines 98-101, Laboratory methods (lines 144-145), two different methods were used for IgG (nephelometry and immunoturbidimetry). Was there any harmonization or validation between these two methods across the long study period? This should be briefly mentioned to assure readers that measurements are comparable over time.*

A2: Please see the answer to Reviewer 1, major comment 3.

*Q3: A major potential confounder for hypergammaglobulinemia is co-infection with Hepatitis C virus (HCV), which is common in PWH and a known cause of high IgG concentration. The authors did state this as a limitation (lines 327-329), but they do not provide any data. Ideally, the prevalence of HCV co-infection (and other chronic infections like Hepatitis B, Syphilis, and Epstein-Barr virus) should be reported for the key groups, especially the high IgG groups and the ART group. If data is available, a sensitivity analysis excluding HCV+ (or for any other chronic infectious disease) individuals would greatly strengthen the conclusion that the hypergammaglobulinemia is HIV-driven. If no data is available, this must be stated as a significant limitation.*

A3: Thank You for this important comment. Information on hepatitis C co-infection has now been added to Methods and Results sections (lines 100-101 and 253-257). We agree that co-infection with hepatitis C could represent a potential confounder. As suggested, we have therefore collected data on hepatitis C status and performed a sensitivity analysis excluding individuals with positive or unknown hepatitis C status. Significantly elevated IgG levels remained in all HIV-positive groups, strengthening the conclusion that the observed hypergammaglobulinemia is primarily driven by HIV. These findings are now commented on in the Discussion (lines 337-341). Unfortunately, we do not have corresponding data on potential co-infections, such as EBV, syphilis or hepatitis B, and this limition has been noted in the manuscript (lines 377-379).

*Minor comments*

*Q1: In line 167. The authors mention that ‘Partial correlation was performed when deemed appropriate.’ This is too vague. The authors need to specify what variables were adjusted for in the partial correlations (e.g., in Fig 3, were correlations adjusted for age, sex?).*

A1: We have now specified in the Statistical methods section (lines 169-170) the conditions under which partial correlation for age was applied. This information was already stated in the legend for Figure 3, but has now been completed in the text for completeness.

*Q2: 2. In the results section, Table 1, the female percentage in the ‘With PrEP’ control group is 0% (0/45). This is a significant demographic difference from the ‘Without PrEP’ group (42.3% female) and the PWH groups. This should be noted in the text, since sex is later shown to have an influence on IgG levels.*

A2: We agree that women contribute a smaller proportion of both the HIV-negative control group and the HIV-positive groups. We have commented that no women were included in the HIV-negative controls with PrEP (lines 180-181) and in the legend of Figure 2 (lines 251-252).
