# Supplementary material for: Hypergammaglobulinemia in treated and untreated people with HIV
Source: PLoS One. 2026 May 21;21(5):e0349405. doi: 10.1371/journal.pone.0349405 (PMC13193345; doi:10.1371/journal.pone.0349405)
Supplement: Attachment — Submitted filename: Review comments.docx [file pone.0349405.s005.docx]

**Peer Review Report**

**Manuscript Title:** Hypergammaglobulinemia in treated and untreated people with HIV

I thank the editorial team for the opportunity to review this revised manuscript. I commend the authors for their thoughtful engagement with the previous review comments; the amendments have certainly strengthened the paper. However, to ensure the highest level of clarity and rigor, I have a few remaining points that require the authors' attention before the manuscript can be considered for acceptance.

**Specific Comments:**

1. Clarity of Statistical Reporting (Major Point): In Figure 3, the presentation of the correlation coefficients requires revision for consistency and clarity. Specifically, the notation for the correlation coefficients (rp) should be standardized to use a decimal point, not a comma. For instance:

- In Fig 3C, please clarify whether the values are `rp = 0.076, p = 0.102` and `rp = 0.074, p = 0.263`.

- Similarly, in Fig 3E, the value should be presented as `rp = -0.277, p < 0.001`.

Ensuring this standardized formatting is crucial for unambiguous data interpretation.

2. Discussion of Demographic Limitations (Major Point): The authors have appropriately addressed several reviewer comments and have noted the male predominance within the Swedish epidemic cohort. However, a critical demographic imbalance persists between the control groups that warrants explicit acknowledgment as a study limitation.

- The ‘With PrEP’ control group is exclusively male (0% female, 0/45), which stands in stark contrast to the 'Without PrEP' control group (42.3% female) and the cohorts of people with HIV (PWH).

- This significant difference introduces a potential source of demographic bias that could influence the comparative analyses of immunoglobulin levels. I strongly recommend that the authors explicitly add a statement to the Discussion or Limitations section acknowledging this imbalance. A cautionary note on how this might affect the generalizability or interpretation of the findings related to the PrEP-naïve control group would significantly strengthen the manuscript's scientific rigor and transparency.
