# Supplementary material for: Hypergammaglobulinemia in treated and untreated people with HIV
Source: PLoS One. 2026 May 21;21(5):e0349405. doi: 10.1371/journal.pone.0349405 (PMC13193345; doi:10.1371/journal.pone.0349405)
Supplement: Attachment — Submitted filename: Response to reviewer - resubmission 2.docx [file pone.0349405.s006.docx]

**Response to reviewer 3**

“Hypergammaglobulinemia in treated and untreated people with HIV”

First, we sincerely thank the reviewer for once again carefully evaluating our manuscript. We appreciate the constructive comments and have revised the manuscript accordingly. Please find our detailed responses to each comment below.

*Major comment*

*Q1: Clarity of Statistical Reporting (Major Point): In Figure 3, the presentation of the correlation coefficients requires revision for consistency and clarity. Specifically, the notation for the correlation coefficients (rp) should be standardized to use a decimal point, not a comma. For instance: In Fig 3C, please clarify whether the values are `rp = 0.076, p = 0.102` and `rp = 0.074, p = 0.263`. Similarly, in Fig 3E, the value should be presented as `rp = -0.277, p < 0.001`. Ensuring this standardized formatting is crucial for unambiguous data interpretation.*

A1: Thank you for pointing this out. The comma has been corrected to a decimal point in the revised Figure 3.

*Q2: Discussion of Demographic Limitations (Major Point): The authors have appropriately addressed several reviewer comments and have noted the male predominance within the Swedish epidemic cohort. However, a critical demographic imbalance persists between the control groups that warrants explicit acknowledgment as a study limitation.*

*- The ‘With PrEP’ control group is exclusively male (0% female, 0/45), which stands in stark contrast to the 'Without PrEP' control group (42.3% female) and the cohorts of people with HIV (PWH).*

*- This significant difference introduces a potential source of demographic bias that could influence the comparative analyses of immunoglobulin levels. I strongly recommend that the authors explicitly add a statement to the Discussion or Limitations section acknowledging this imbalance. A cautionary note on how this might affect the generalizability or interpretation of the findings related to the PrEP-naïve control group would significantly strengthen the manuscript's scientific rigor and transparency.*

A2: We agree that the gender imbalance in the HIV-negative control group on PrEP represents a potential limitation of the study. To further address this, we have added statements in the Discussion section (lines 332-334 and 380-382), acknowledging this imbalance and its potential influence on the generalizability of the findings.
