# Supplementary material for: Hypergammaglobulinemia in treated and untreated people with HIV
Source: PLoS One. 2026 May 21;21(5):e0349405. doi: 10.1371/journal.pone.0349405 (PMC13193345; doi:10.1371/journal.pone.0349405)
Supplement: Attachment — Submitted filename: Comment.docx [file pone.0349405.s007.docx]

I thank the authors for the thoughtful and comprehensive responses to the reviewers’ comments. The revisions have substantially strengthened the manuscript in terms of clarity, rigor, and overall presentation. The authors have adequately addressed the concerns raised, and the manuscript has improved considerably as a result.

I recommend acceptance of the manuscript for publication.
